# The Investigation of Gas Distribution Asymmetry Effect on Coriolis Flowmeter Accuracy at Multiphase Metering

**DOI:** 10.3390/s22207739

**Published:** 2022-10-12

**Authors:** Evgeniia Shavrina, Yan Zeng, Boo Cheong Khoo, Vinh-Tan Nguyen

**Affiliations:** 1 Department of Mechanical Engineering, National University of Singapore, Singapore 119260, Singapore; 2 National Metrology Centre, Singapore 138634, Singapore; 3 Institute of High Performance Computing, Singapore 138632, Singapore

**Keywords:** Coriolis flowmeter, multiphase flow, gas distribution asymmetry, numerical simulation

## Abstract

Multiphase flows are encountered in various industries, and the Coriolis flowmeter (CFM) is considered a high potential flowmeter for the metering of these flows. However, the decoupling effect and asymmetrical gas distribution in a CFM might decrease the accuracy of its multiphase flow metering The asymmetry of gas distribution in a CFM and its influence on the metering accuracy have only been qualitatively investigated in a few studies. The present paper quantitatively describes the gas distribution asymmetry in several CFMs under different flow conditions by numerical simulation. The simulation methodology is developed and validated by a results comparison with a conducted experiment and published data for bubbly, stratified and transitional flow regimes. U-shaped and triangle-shaped CFMs of different diameters are investigated at different gas volume fractions and flow rates. It is shown that the increase in the gas volume fraction and the reduction in the mixture flow rate lead to the increase in the gas distribution asymmetry. The strong correlation between the gas distribution asymmetry and the experimentally observed CFM error is demonstrated. The correction of the CFM error is proposed based on this correlation allowing the metering error to be decreased from 34% to 10% for the investigated conditions.

## 1. Introduction

Nowadays, multiphase fluids are one of the most common fluids, and may be found in medical equipment, and in the aerospace, energy storage and other industries. For example, promising energy storage liquids and fuels, i.e., hydrogen, liquefied natural gas and liquid air, often contain gaseous components, i.e., bubbles, due to their cryogenic nature [1,2,3]. At the same time, emulsions and foams are indispensable components in food and beverage production [4]. Moreover, a bubbly flow is a common phenomenon in medical ultrasound applications, including drug delivery [5] and ultrasonic imaging [6]. Hence, the accurate metering of multiphase flows is deemed necessary for further development in the discussed and some other essential fields.

A Coriolis flowmeter (CFM) is considered one of the promising tools in multiphase fluid metrology [7,8,9]. This is explained by the fact that the costs of traditional multiphase metering, which is based on the multiphase separation, are not acceptable in many applications [10]. This flowmeter consists of the sinusoidal oscillating tube or tubes, the deformation of which depends on the mass flow rate of the fluid flowing through [7]. The tube deformation is read by the sensors and characterized as the time shift, which is the difference in time when the inlet and outlet sides of the flowmeter tube are returning to the initial position. This operational principle requires an absolute symmetry of the CFM tube, which is usually U-shaped or triangle-shaped.

The CFM accuracy is supposedly aimed at being higher than 99.9% for multiphase fluids, including viscous fluids, emulsions, gases and bubbly flows [11]. However, several studies have shown that the gas in the flow may cause a drop in the CFM accuracy [7,12,13,14,15,16,17,18,19,20]. The CFM error at multiphase metering is assumed to be primarily caused by the decoupling effect according to several studies [16,17,21]. The decoupling effect is thought to be caused by the relative motion between bubbles and liquid in the direction of the flowmeter tube oscillation [21]. This relative motion acts in the same way as damping and decreases the tube oscillation amplitude. Consequently, this leads to a decrease in the amplitude, reduces the flowmeter reading and causes a negative error. The CFM metering error has been quantified based on the decoupling effect as follows [22,23,24]

(1) $$\varepsilon_{d}=-\frac{2\alpha }{1-\alpha },$$

where *α* is the gas volume fraction, and $\varepsilon_{d}$ is a CFM mass flow rate error estimation based on decoupling effect.

At the same time, some other studies have shown that Equation (1) overestimates the negative CFM error and is not applicable in some cases at all [15,23,25,26,27]. Sultan [23] shows that the negative error is overestimated for most of the investigated experimentally cases. Weinstein [15], Liu et al. [25] and Wang et al. [26] report positive CFM errors for some cases of multiphase metering. In addition, Wang et al. [26] and Liu et al. [25] state that the CFM error does not follow Equation (1) with the increase in the gas volume fraction. Pushnov confirmed this fact for different operational pressures. Henry et al. [27] present the experimental data showing that the CFM error depends not only on the GVF but also on the flow rate, indicating the limitations of Equation (1). Finally, Wang et al. [28] also demonstrate a positive CFM error during the measurement of the gas–liquid mixture in their experimental study.

Pushnov [29] suggests that the non-uniform gas distribution along a curved CFM tube may cause an unpredictable CFM error. This gas distribution asymmetry deteriorates the symmetry of the tube mass, which is the foundation of the CFM operation [30]. According to the experimental study conducted by Enz et al. [30], if the mass center of a CFM tube is shifted to the CMF inlet, the CFM error is positive or vice versa. Weinstein [15] experimentally demonstrated that a tubes-down CFM orientation causes gas accumulation in the inlet side of the CFM tube and a positive CFM error. According to the same study, the tubes-up orientation is associated with the gas accumulation in the outlet side of the flowmeter tube and negative error [15]. However, these qualitative observations were only made by Weinstein due to the limitations of the experimental study. At the same time, according to Wang et al. [26], both positive and negative errors may be observed for the CFM in the same orientation. However, the gas distribution itself was not studied in this paper [26]. Therefore, while the importance of the gas distribution asymmetry is indicated in some studies, it has not been investigated quantitatively. Since it may resolve the limitations of the CFM error explanation by the decoupling effect, the gas distribution asymmetry is investigated in the present study.

The present paper aims to describe and quantify the gas distribution asymmetry in different CFMs by computational simulation at various flow conditions. Additionally, the correlation between the numerically calculated gas distribution asymmetry and the experimentally observed flowmeter error is demonstrated. Finally, the CFM error correction, which is based on the gas distribution asymmetry effect, is presented. It is compared with the previously used correction, based on the decoupling effect and the experimentally observed non-corrected error. Therefore, it is hoped that this study will further improve our understanding of the causes behind the CFM multiphase metering error and provide a correction methodology for the metering accuracy improvement for multiphase flow metering.

## 2. Methods

### 2.1. Investigated Coriolis Flowmeters

Three different Coriolis flowmeters (CFMs) by Emerson are investigated in the present study by numerical and experimental approaches. Firstly, the performances of CMF200 and CMF050 at multiphase metering are experimentally investigated. Secondly, the multiphase flows inside of these CFMs are analyzed by conducting the computational simulation. Finally, the numerical simulation of D300, which has been experimentally studied by Sultan [23], is conducted.

**Figure 1:**
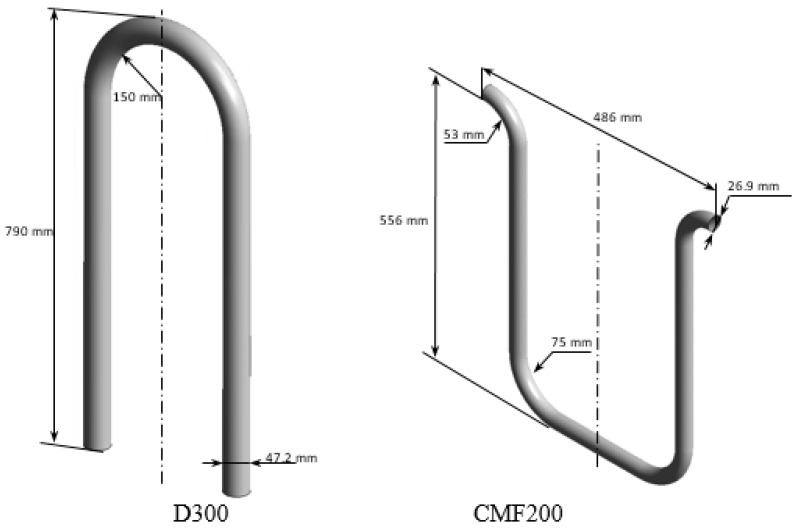

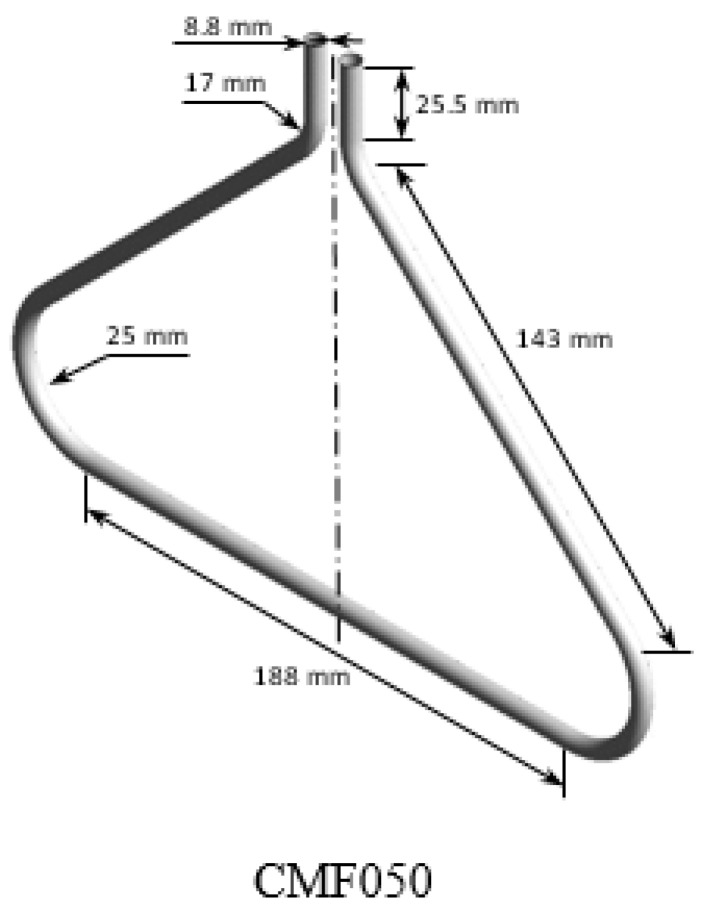
The dimensions of the modelled CFMs tubes.

It should be noted that the investigated flowmeters are installed in different orientations: D300 is in the down-tube orientation, while CMF200 and CMF050 are in the up-tube position, as demonstrated in Figure 1. The geometrical dimensions of D300 flowmeter tube are described by Sultan [23] and presented in Figure 1. However, the detailed geometrical dimensions of CMF200 and CMF050 are not specified by the manufacturer. These dimensions are obtained from the general dimensions of the CMF200, which were refined by multiobjective genetic algorithm optimization [31,32] so that the natural frequency of the flowmeter tube corresponds to the known CMF200 natural frequency of 93.933 Hz [33]. Since the natural frequency is dependent only on the material, which is specified by manufacturer, and the tube dimensions, it may be assumed that the shape of the CFM tube is determined accurately. The obtained dimensions of the modelled CMF200 and CMF050, are presented in Figure 1.

### 2.2. Methodology of Experimental Study of CFM Operation at Multiphase Metering

**Figure 2:**
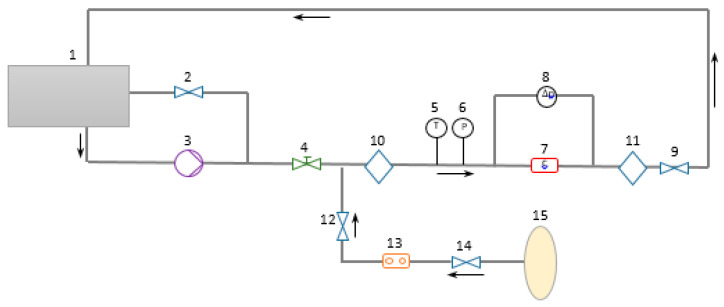
The experimental setup scheme. 1—water tank, 2—by-pass valve; 3—water pump; 4—control valve; 5—temperature sensor; 6—pressure sensor; 7—CFMs; 8—differential pressure sensor; 9—fine-tune valves; 10—upstream optical window; 11—downstream optical window; 12—gas injection valve; 13—gas flowmeter; 14—regulator; 15—air cylinder.

The experimental study is conducted to determine the error of the multiphase metering by a CFM at different flow rates and gas volume fractions. The experimental setup, which is shown in Figure 2, is established in National Metrology Centre (NMC), A*STAR Research Entities, Singapore.

**Figure 3:**
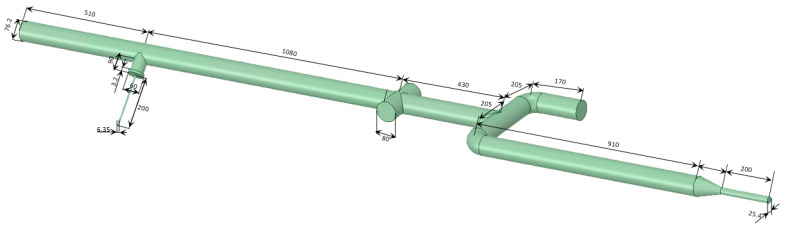
The dimensions of experimental setup before the CFMs.

Firstly, water is pumped from storage tank (1) to the pipeline by pump (3), Figure 2. The flow in the pipe is controlled by valve (4), which is demonstrated in Figure 2. The temperature and gauge pressure values are measured by sensors (5) and (6), respectively, Figure 2. After this, the water flow goes through the Coriolis flowmeter (7), where the temperature, density and mass flow rate are determined. The U-shaped CFMs (CMF200 and CMF050 by Emerson) are investigated in the present study. These two flowmeters work in the ultra-low (CMF200) or low frequency (CMF050) to increase the measurement accuracy by minimizing the resonance effect [13]. The water flow rate is controlled by a group of fine-tune valves (9). At the same time, compressed air is supplied to the system from the gas cylinder (15) through valves (12) and (14) by using the gas flowmeter (13) by Dwyer to control the gas flow rate, Figure 3. This ensures the multiphase flow in the system. Finally, 80 mm diameter optical windows (10) and (11) provide a visualization of the multiphase flow patterns before and after the CFM. The setup dimensions before the CFM are presented in Figure 3.

The different gas volume fractions (GVFs) at the CFM inlet ($\alpha_{inlet}$) and water flow rates are experimentally investigated in the present study for CMF200 and CMF050 as demonstrated in Table 1. The GVF value at the inlet of CFM is calculated following Equation (2) as a ratio between gas and water volume flow rates, which are experimentally measured, following the studies by Sultan [23] and Athanase [33]. The used gas flowmeter was Dwyer Rate-Master RMA-21-SSV Flow Meter-1-10 LPM Air Range model with the metering error of 4%. The single-phase water flow rate was measured by CMF200 or CMF050 depending on the investigation case with the error of 0.05%. The flow regime, which is indicated in Table 1, is observed in the optical windows of the experimental setup. It should be noted that the transitional flow regime refers to the stratified flow before the CFM and bubbly flow after the CFM. The selected parameters allow an efficient investigation of GVF and mixture flow rate influences on the CFM accuracy. Here

(2) $$\alpha_{inlet}=\frac{VFR_{g}}{VFR_{g}+VFR_{w}}\cdot 100\%$$

is the GVF at the inlet of the CFM tube, where $VFR_{g}$ is the gas volume flow rate and $VFR_{W}$ is the water volume flow rate.

The relative error of the multiphase metering by CFMs is quantified in the present experimental study following Equation (3). This is in accordance with the best practices for the analysis of the flowmeter operation at multiphase flow as demonstrated by TUV NEL [34] and some other studies of CFM error at multiphase metering [28,35].

(3) $$\begin{array}{c} \varepsilon_{e}=\frac{MFR_{m}-(MFR_{g}+MFR_{w})}{MFR_{g}+MFR_{w}}\cdot 100\%;\\ MFR_{g}=\rho_{g}\cdot VFR_{g}, \end{array}$$

where $MFR_{m}$ is the measured mass flow rate at multiphase flow regime, $MFR_{g}$ is the gas mass flow rate measured by gas flowmeter, $VFR_{g}$ is gas volume flow rate measured by the gas flowmeter, $\rho_{g}$ is the gas density and $MFR_{W}$ is the water mass flow rate measured by CFM before the gas introduction.

### 2.3. Methodology of Computational Study of Gas Distribution Asymmetry in CFM

Numerical simulations were conducted to investigate the performance of CFM models and obtain the insight on the CFM operation. In this work, a set of transient simulations was performed using multiphase flow models in the commercial software ANSYS CFX. Air was modelled as compressible gas, while water was modelled as the incompressible liquid following the experimental study conditions. The Reynolds Stress turbulence model was selected for all conducted simulations of the flows through the selected CFMs. This model was selected as its high accuracy, above 95%, was confirmed by Shavrina et al. [36] for the simulation of the CFM operation at different flow rates.

As highlighted above, three flow regimes are investigated in the present study, namely: stratified, bubbly and transitional. These regimes are modelled by different modifications of Eulerian–Eulerian multiphase model [37] following the best practices to achieve a sufficiently accurate solution and maintain the acceptable computational time. For the stratified flow regime, we employ the Volume of Fluid (VOF) modification of Eulerian–Eulerian model [37]. This modification is selected to describe the sharp interface between gas and liquid phases, which is observed in stratified flow regimes. Since the conducted simulation is transient, a compressive transient scheme is used for the volume fraction equations. The surface tension was assumed to be equal to 72 mN/m in accordance with the data by Vargaftik et al. [38]. Since the transitional regime is a combination between bubbly and stratified flow regimes, it is modelled following VOF as well. In this VOF approach, all phases are modelled as a mixture governed by the momentum conservation

(4) $$\frac{\partial }{\partial t}\left(\rho \vec{v}\right)+\nabla \cdot \left(\rho \vec{v}\vec{v}\right)=-\nabla p+\nabla \cdot \left[\mu \left(\nabla \vec{v}+\nabla {\vec{v}}^{T}\right)\right]+\rho \vec{g},$$

where ρ is the density, *t* is the time, *μ* is the dynamic viscosity, *p* is the pressure, *v* is velocity and $\vec{g}$ is the acceleration due to the gravity. All quantities are for the mixture.

For the bubbly flow regime, the interface between the phases is of less interest. Moreover, the VOF approach requires a fine mesh to resolve the interface between phases, leading to a high computational cost. As such, a classic Eulerian–Eulerian technique is used for the simulation of the bubbly flows instead of VOF approach. In the classic Eulerian–Eulerian approach, the separate momentum equations are solved for each of the phases as follows

(5) $$\begin{array}{c} \frac{\partial }{\partial t}\left(\alpha_{q}\rho_{q}\vec{v_{q}}\right)+\nabla \cdot \left(\alpha_{q}\rho_{q}\vec{v_{q}}\vec{v_{q}}\right)=\\ =-\alpha_{q}\nabla p+\nabla \cdot \overset{\equiv }{\tau_{q}}+\alpha_{q}\rho_{q}\vec{g}+\sum_{p=1}^{n}(K_{pq}\left(\vec{v_{p}}-\vec{v_{q}}\right))+{\vec{F}}_{nd}, \end{array}$$

where *q* is the fluid phase, $\alpha_{q}$ is the gas volume fraction of the *q*th phase, ${\vec{F}}_{nd}$ is the non-drag forces, $\overset{\equiv }{\tau_{q}}$ is the *q*th phase stress–strain tensor and $K_{pq}$ is the interphase momentum exchange coefficient.

The bubbles’ coalescence is modelled by MUSIG approach [37] for the investigation of the bubbly flow regimes. The drag force is modelled according to Izhii Zuber model [37]. The simulated non-drag forces include: wall lubrication force by Frank model [39], lift force by Tomiyama [40], turbulent dispersion force by Favre model [41] and virtual mass force [42].

**Figure 4:**
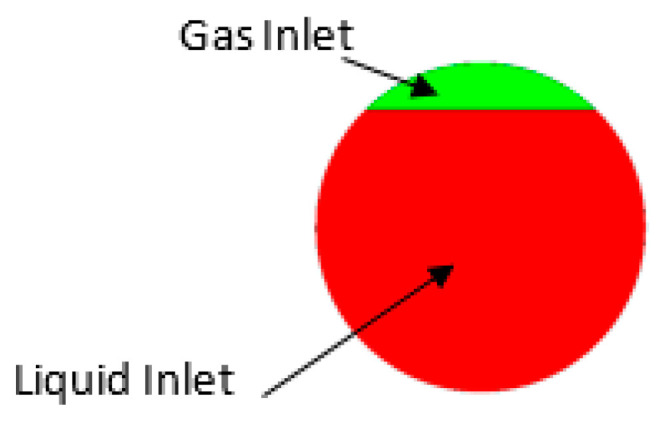
A typical liquid hold-up inlet boundary condition for the stratified flow regime simulation of CFM operation (9% GVF, 60.8 kg/min).

The numerically investigated operation conditions are described in Table 2. Following the experimental conditions, it is assumed that the CFM tube is filled with water as initial condition. The uniform bubble distribution at the inlet of CFM tube was modelled for the bubbly flow following the data provided by Sultan [23]. The mean bubble diameter is assumed to be equal to 0.8 mm for D300 and 0.1 mm for CMF200 based on the analysis of the pictures reported by Sultan [23] for D300 and the observations in the conducted experimental study for CMF200. Since VOF modification is used for the stratified flow, the liquid hold-up is indicated at the inlet of the CFM tube in the stratified flow simulations following the observation in optical windows in the conducted experimental study. This boundary condition is created by dividing the inlet into two parts, of which areas’ ratio is equal to GVF indicated in Table 2, as demonstrated in Figure 4. In addition, the mass flow rates for each phase, which are determined by experimental conditions, Table 1, are used as boundary conditions for the stratified flow simulations.

**Figure 5:**
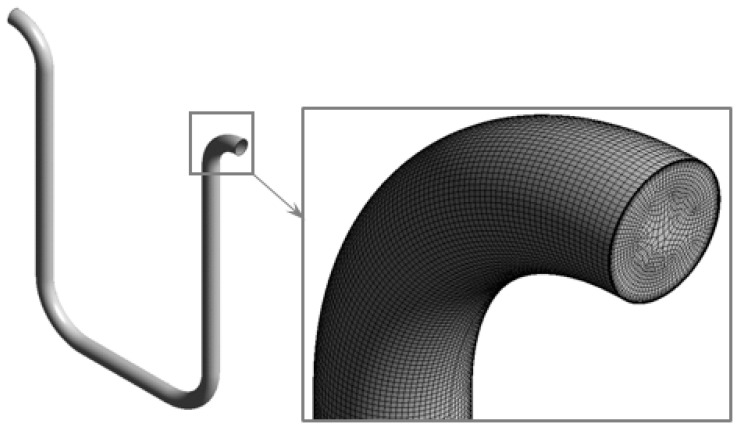
Typical space discretization for the computational simulation.

The CFM geometry is discretized by hexahedral elements in the computational simulation. The average size of the element is selected following the study by Shavrina et al. [36] to ensure the accuracy of the modelling. The inflation layer near the wall is described in such a way to ensure that y+ value is less than one. The typical space discretization is presented in Figure 5. The mesh sensitivity study was conducted for each of investigated flowmeters, showing that the change in the calculated gas distribution asymmetry with a change in the mesh elements size is less than 2%. Hence, there are 2,564,854, 3,478,562 and 5,085,388 mesh elements for CMF200, D300 and CMF050 models, respectively.

**Figure 6:**
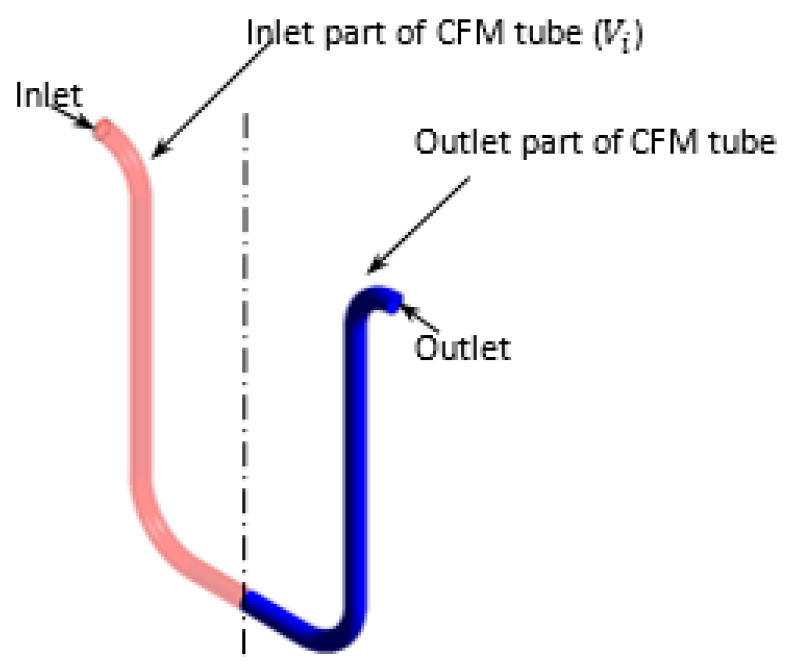
The parts of a CFM tube, which are used for the characterization of gas distribution asymmetry.

The objective of the conducted computational analysis is the quantification of the gas distribution asymmetry. This variable is characterized as a difference between the volume-weighted gas volume fractions (GVFs) in the inlet part ($\gamma_{i}$) and the outlet part ($\gamma_{o}$) of the CFM tube as shown in Equation (6). These parts are the volumes of the CFM tube, which are separated by the symmetry plane, as highlighted by different colors on Figure 6 on CMF200 as an example. Here

(6) $$\gamma_{d}=\gamma_{i}-\gamma_{o}=\frac{\int \alpha dV_{i}}{V_{i}}-\frac{\int \alpha dV_{o}}{V_{o}}$$

is the difference between volume-weighted GVFs at the inlet ($\gamma_{i}$) and outlet part ($\gamma_{o}$) of the CFM tube, where $V_{i}$ is the volume of the inlet part of the tube, and $V_{o}$ is the volume of the outlet part of the tube.

## 3. Results

### 3.1. Validation of Simulation Methodology

The validation of multiphase simulation methodology is conducted to ensure the reliability of the calculated flow pattern and, consequently, the gas distribution asymmetry in a CFM. It is also necessary to ensure the reliability of the described simulation methodology. This is achieved by the comparison of numerical results with the experimental data. As indicated in Table 2, stratified, transitional and bubbly flow regimes are investigated in the present study. Since the transitional regime is a combination of stratified and bubbly flow regimes, only validations for stratified and bubbly regimes are conducted.

#### 3.1.1. Validation of Stratified Flow Regime Simulation Methodology

**Figure 7:**
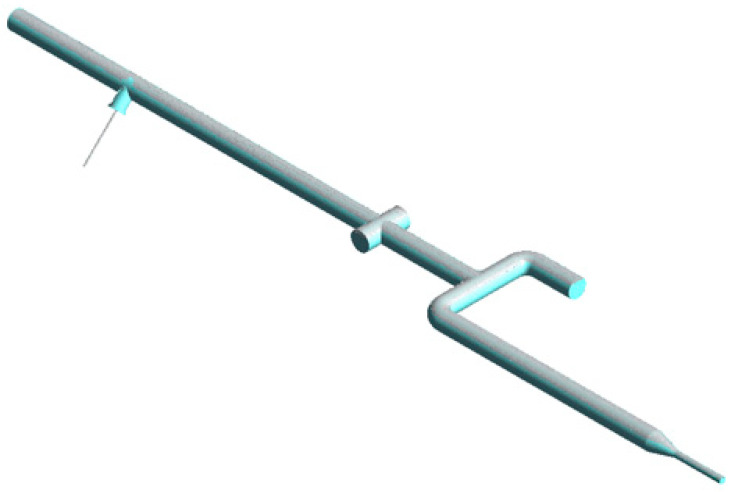
The gas distribution in the experiment study setup before the CFMs.

Firstly, the simulation results are compared with the experimental data for the stratified flow regime. Since the measurement of the flow inside of the CFM is not feasible, the simulation of the flow in the experiment setup before the CFM is conducted, the dimensions of which are demonstrated in Figure 3. The simulation is conducted for a 60 kg/min water flow rate and a 6 l/min gas flow rate. The calculated gas distribution is presented in Figure 7.

**Figure 8:**
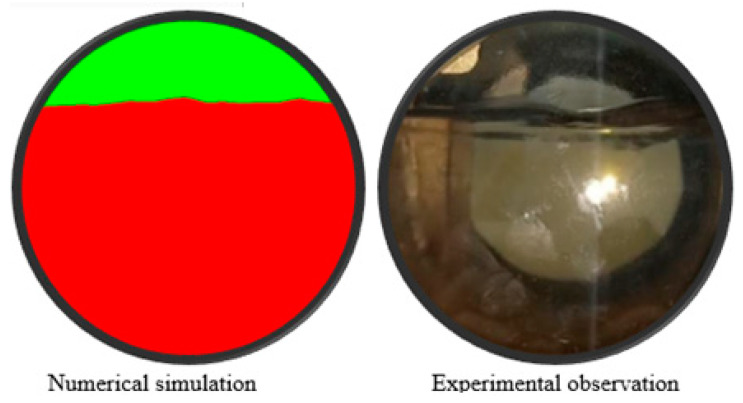
The flow pattern in the optical window according to the experimental observation and numerical simulation.

The liquid hold-up is a key parameter for the characterization of the stratified flows [43,44,45,46]. In the present study, it is calculated following Equation (7) for the experimental observations in the optical window and for the liquid hold-up obtained by numerical simulation at the same location, which are demonstrated in Figure 8. The chord of the observed circle segment is measured, and the area, which is occupied by gas, is calculated. The area occupied by the water is calculated as a difference between the observation window area and the gas occupied area. The relative error of the calculated liquid hold-up in comparison with the experimental data is less than 0.5%. Hence, the applied simulation methodology ensures a high accuracy of stratified flow simulation.

(7) $$\eta_{L}=\frac{A_{L}}{A_{L}+A_{G}},$$

where $\eta_{L}$ is the liquid hold-up, $A_{L}$ is the area occupied by liquid and $A_{G}$ is the area occupied by gas.

#### 3.1.2. Validation of Stratified Flow Regime Simulation Methodology

Secondly, the validation of the bubbly flow simulation is conducted by the comparison of the simulation results with the experimental data by Prasser et al. [47], which is often used for the validation of bubbly flow simulations [48,49,50,51,52]. The gas volume fraction distribution in a vertical pipe is investigated in this experimental study [47]. The air–water mixture is observed in the pipe, which has a diameter of 195 mm and length of 8000 mm. The mixture flow rate is equal to 29 kg/s, and the gas volume fraction is equal to 1.8% at the inlet of the pipe. In addition, the bubbles’ diameter distribution is provided by Prasser et al. [47], which is described in Table 3. The GVF distributions at the pipe inlet are assumed to be uniform as it is not supposed to influence the results at a large distance from the inlet, according to Liao et al. [53].

**Figure 9:**
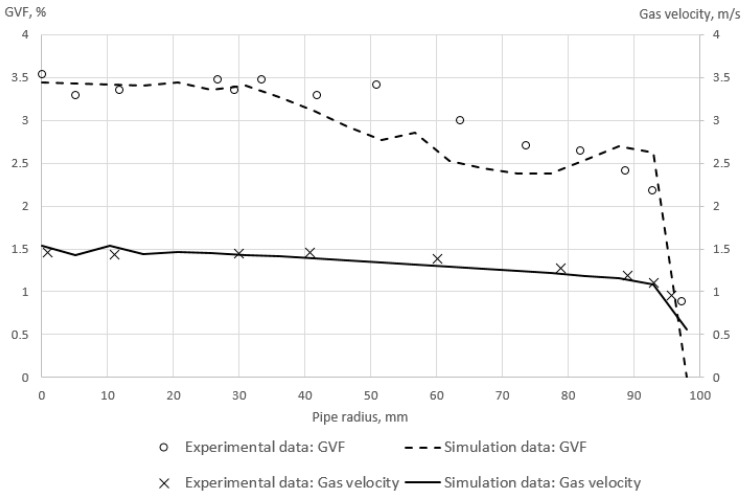
The gas volume fraction distribution and gas velocity across the pipe radius according to the conducted simulation and experimental study by Prasser et al. [47].

The gas volume fraction distribution across the tube radius is calculated following the described methodology and compared to the experimentally reported values at L/D = 40, where L is a coordinate along the pipe axis and D is the pipe diameter. This comparison is presented in Figure 9. The maximum absolute error of the calculated GVF in comparison with the experimental data by Prasser et al. [47] is less than 0.75. The observed error might be explained by an experimental error in the GVF measurement, which achieves 1% of GVF [47,54]. Hence, it may be concluded that the described methodology is suitable for the accurate simulation of gas distribution at bubbly flow.

#### 3.1.3. Validation of CFM Tube Oscillation Neglect Assumption

**Figure 10:**
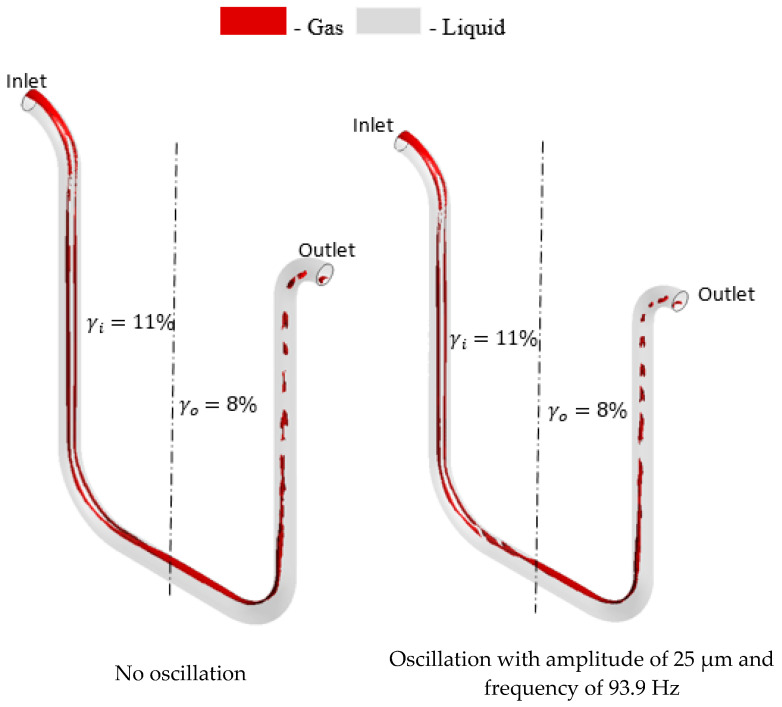
The gas distribution in flowmeter tube with and without oscillation at 149 kg/min mixture flow rate, 9% GVF at the inlet, CMF200.

Finally, the comparison of the gas distributions in oscillating and non-oscillating CFM tubes is demonstrated in Figure 10. It may be noted that the oscillation parameters are selected in accordance with the study conducted by Athanase [33]. The calculated difference between the gas distribution asymmetries in oscillating and non-oscillating tubes is less than 1%. Hence, the oscillation of the flowmeter tube is neglected in the present study for simplicity without affecting the accuracy of the measured gas distribution asymmetries.

Overall, the validation of the multiphase simulation methodology is conducted for bubbly and stratified flow regimes. The comparison of the computational results with experimental data confirms a high accuracy of numerically obtained gas distribution. In addition, it is demonstrated that the CFM oscillation may be neglected for the investigation of the gas distribution asymmetry to decrease the computational costs of the simulations without a significant accuracy loss.

### 3.2. The Gas Distribution Asymmetry in the CFM Tube at Different Conditions

**Figure 11:**
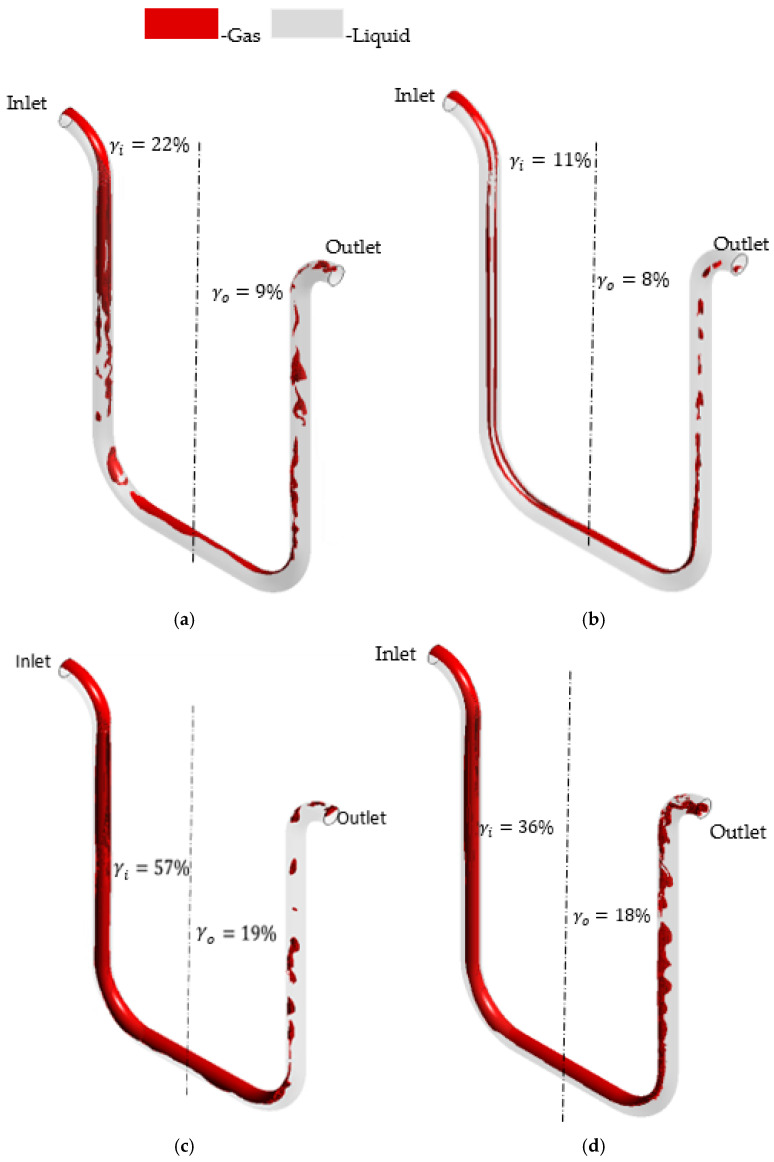

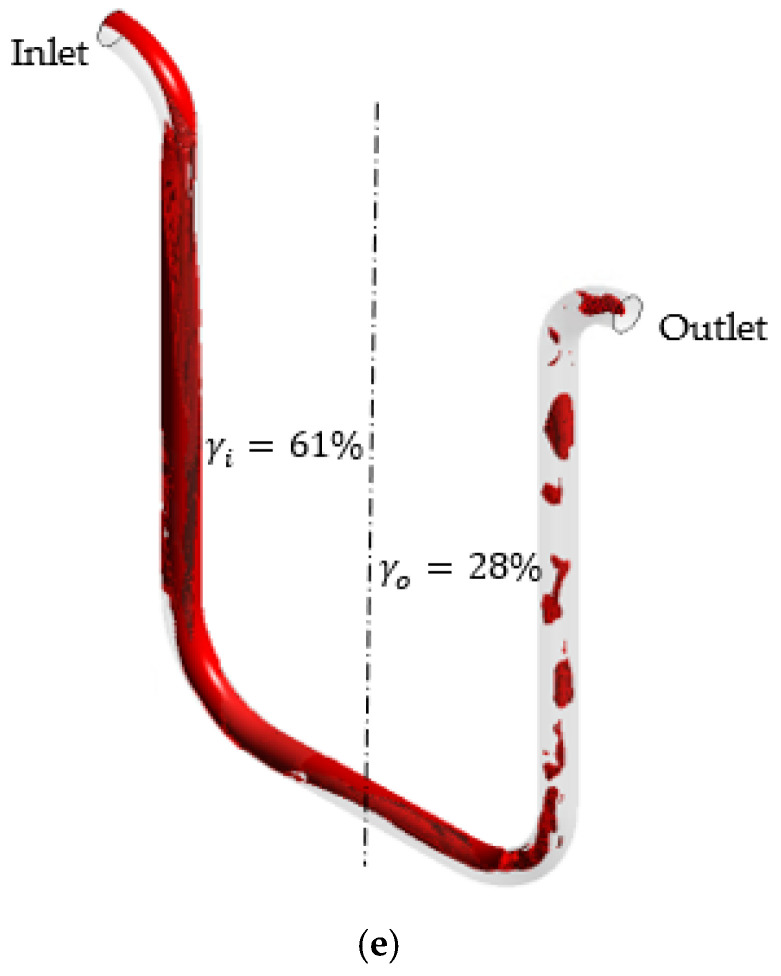
The gas distribution in flowmeter tube at different flow conditions obtained by numerical simulation at stratified and transitional flow regimes for CMF200. (**a**) 60.8 kg/min mixture flow rate, 9% GVF at the inlet, 13% gas asymmetry $\left(\gamma_{d}\right)$, CMF200. (**b**) 149.0 kg/min mixture flow rate, 9% GVF at the inlet, 3% gas asymmetry $\left(\gamma_{d}\right)$, CMF200. (**c**) 60.8 kg/min mixture flow rate, 16% GVF at the inlet, 38% gas asymmetry $\left(\gamma_{d}\right)$, CMF200. (**d**) 100.0 kg/min mixture flow rate, 16% GVF at the inlet, 18% gas asymmetry $\left(\gamma_{d}\right)$, CMF200. (**e**) 60.8 kg/min mixture flow rate, 13% GVF at the inlet, 33% gas asymmetry $\left(\gamma_{d}\right)$, CMF200.

The gas distribution asymmetry in the CFM tube is investigated at different operating conditions by numerical simulation. Firstly, the gas distributions in the CMF200 tube are presented in Figure 11 for stratified and transitional flow regimes. The demonstrated gas distribution patterns are determined by buoyancy and centrifugal forces similarly to a gas–liquid pattern observed in any U-shaped tube. The centrifugal force draws the gas to the center of the U-turn of the CFM. Hence, this force brings the gas closer to the symmetry plane in the vertical sections of the tube, Figure 11. The centrifugal force is aligned with the buoyancy force in the horizontal section of the CFM tube, leading to the gas aggregation in the upper half of this tube section, Figure 11. The buoyancy force acts in the opposite direction with the momentum in the vertical inlet part of the CFM tube. At the same time, the directions of buoyancy force and momentum coincide in the vertical outlet part of the CFM tube, accelerating the gas in this part of the tube. This leads to gas accumulation in the vertical inlet part of the CFM. This qualitatively agrees with observations of gas–liquid mixture flows in U-shaped tubes, which are oriented in the same way and have been experimentally investigated by Deng at al. [55] and Weinstein [15].

**Figure 12:**
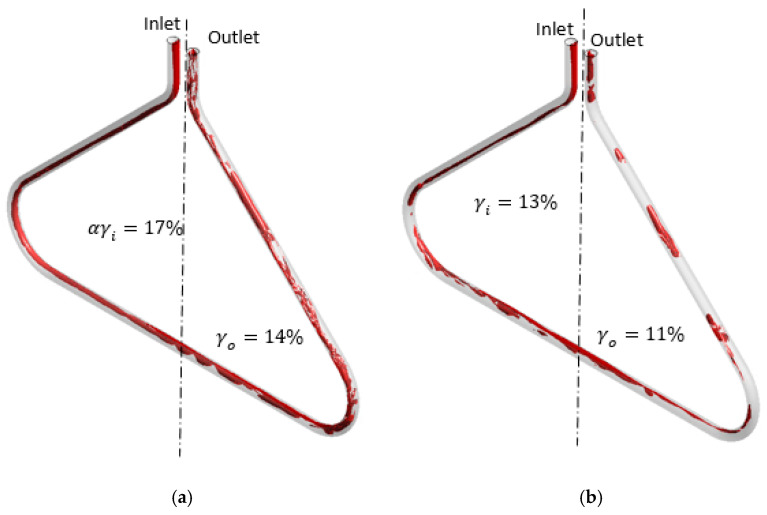
The gas distribution in flowmeter tube at different flow conditions obtained by numerical simulation at stratified and transitional flow regimes for CMF050. (**a**) 40.0 kg/min mixture flow rate, 15% GVF at the inlet, 3% gas asymmetry $\left(\gamma_{d}\right)$, CMF050. (**b**) 40.0 kg/min mixture flow rate, 10% GVF at the inlet, 2% gas asymmetry $\left(\gamma_{d}\right)$, CMF050.

A similar gas distribution pattern is observed in CMF050. As demonstrated in Figure 12, the centrifugal force also draws the gas to the centre of the triangle shape of the CFM: the gas is attracted to the symmetry plane in the diagonal sections of the tube. The centrifugal force is aligned with the buoyancy force in the horizontal section of the tube, leading to gas accumulation in the upper half of this tube section similarly to CMF200. The relationship between the buoyancy force and the momentum in CMF050 is also similar to this relationship in CMF200. Hence, the gas accumulates more in the inlet part of the CFM.

**Figure 13:**
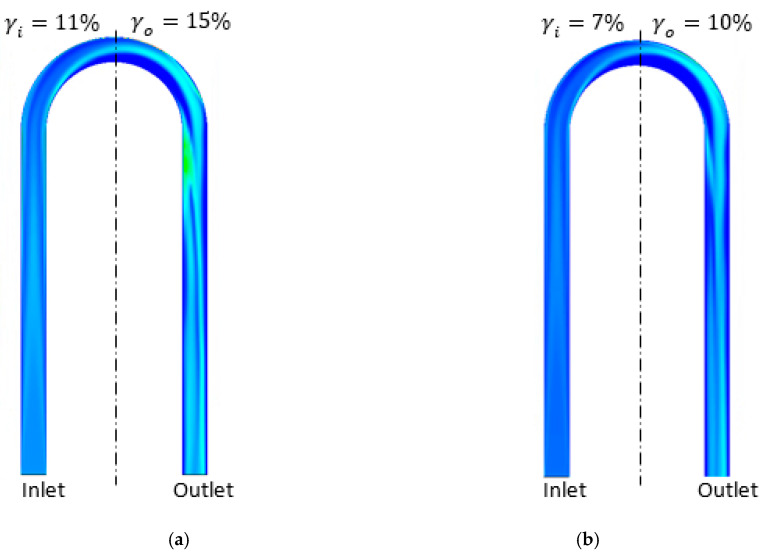

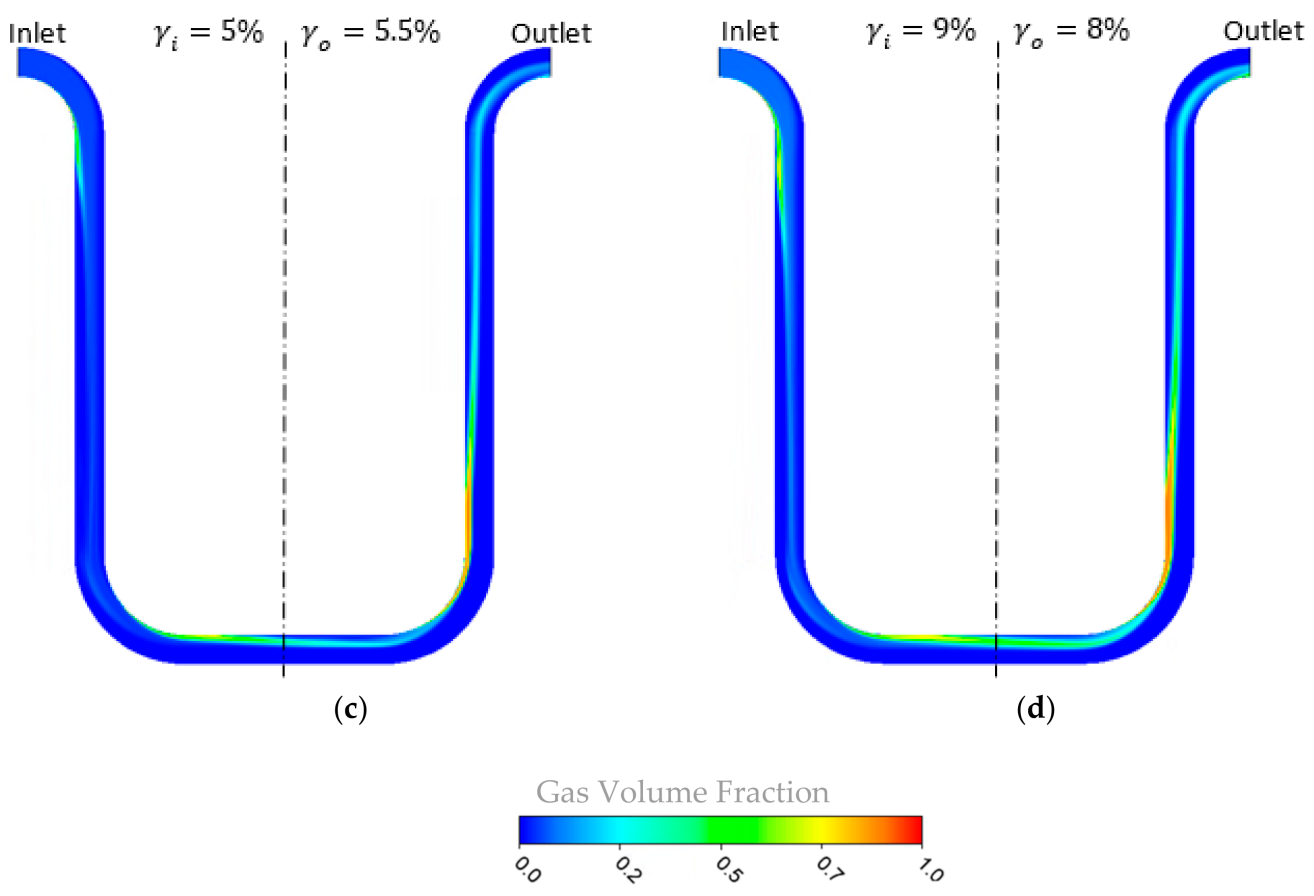
The gas distribution in flowmeter tube at different flow conditions obtained by numerical simulation at bubbly regimes. (**a**) 182 kg/min mixture flow rate, 12% GVF at the inlet, −4% gas asymmetry $\left(\gamma_{d}\right)$, D300. (**b**) 182 kg/min mixture flow rate, 8% GVF at the inlet, −3% gas asymmetry $\left(\gamma_{d}\right)$, D300. (**c**) 300 kg/min mixture flow rate, 5% GVF at the inlet, −0.5% gas asymmetry $\left(\gamma_{d}\right)$, CMF200. (**d**) 300 kg/min mixture flow rate, 8% GVF at the inlet, 1% gas asymmetry $\left(\gamma_{d}\right)$, CMF200.

In addition, the bubbly flow regime is investigated for CMF200 and D300, following the methodology for a classic Eulerian–Eulerian approach. It should be highlighted that D300 is oriented in a tubes-down position, while CMF200 is installed in a tubes-up orientation. Since D300 is oriented in a tubes-down position, the gas acceleration, which is caused by buoyancy force, acts in the same direction as the flow momentum in the inlet part of the D300 tube. However, the same gas acceleration acts in the opposite direction to the flow in the outlet D300 part. Hence, the gas accumulates more in the outlet part of D300 for the investigated flow rate and GVFs as demonstrated in cases (a) and (b) in Figure 13. This agrees with the experimental observations by Deng et al. [55], who qualitatively demonstrated the same relationship between U-shaped tube orientation and gas accumulation location.

At the same time, the bubbles in CMF200 are too small and the inlet GVF is too low to ensure sufficient buoyancy force to overcome the flow momentum in the inlet vertical part of the CMF200 at 5% inlet GVF. Because of this, the bubbles are distributed almost perfectly following the flow field in CMF200 at 5% GVF, case (c) Figure 13. Because of the separation region in the outlet turn part of the CMF200 tube, the bubbles are slightly accumulated in the outlet part for 5% GVF as demonstrated in case (c) Figure 13. However, since the GVF achieves 8% in case (d) Figure 13, the bubbles’ buoyancy force is able to overcome the momentum in the inlet vertical part of the CFM. Hence, the bubbles aggregate in the inlet part of the CMF200 at 8% GVF, as demonstrated in case (d) Figure 13.

The relationship between the GVF at the inlet and the gas distribution asymmetry is investigated for different flow regimes and CFMs. According to Figure 11, Figure 12 and Figure 13, a higher GVF at the inlet of the CFM ($\alpha_{inlet}$) leads to a higher gas distribution asymmetry ($\gamma_{d}$). For example, as demonstrated in cases (a), (c) and (e) in Figure 11, the growth in the GVF at the CMF200 inlet from 9% to 13% to 16% leads to the increase in the gas asymmetry from 17% to 33% to 38% for the mixture flow rate of 60.8 kg/min. This pattern is also observed for CMF050, as demonstrated in Figure 12, the growth in the GVF from 10% to 15% leads to the asymmetry growth of 1%. The same pattern is observed for the bubbly flow according to Figure 13 for the cases (a) and (b), D300, and the cases (c) and (d), CMF200. This is explained by the fact that a higher inlet GVF ($\alpha_{inlet}$) at the same mixture flow rate is achieved by a higher gas flow rate, which experiences a more significant buoyancy force due to a larger gas mass.

Additionally, the relationship between the gas distribution asymmetry and the mixture flow rate is studied. As demonstrated in Figure 11, the gas asymmetry ($\gamma_{d}$) grows from 3%, case (b), to 18%, case (a), with the decrease in the flow rate from 149.0 kg/min to 60.8 kg/min at 9% GVF at the CMF200 tube inlet. This is consistent with the simulation results for another pair of the flow rates at GVF at the CMF200 inlet of 16%: the gas asymmetry ($\gamma_{d}$) equals 38% and 18% for 60.8 kg/min and 100.0 kg/min, respectively, according to the cases (c) and (d) in Figure 11. Hence, the gas distribution asymmetry grows with the decrease in the mixture flow rate. This is due to the fact that the impact of the buoyancy force increases in the case of the low flow rate in comparison with momentum flux. In general, this agrees with a qualitative study by Weinstein [15], which states that the gas accumulation in one side of the triangle-shaped tube might be eliminated by an increase in the mixture flow rate.

Overall, the gas distribution asymmetry depends on the mixture flow rate, gas volume fraction and CFM type, as demonstrated. The lowest value of the gas distribution asymmetry may be achieved by a low GVF at the inlet and a high mixture flow rate. This is explained by the influence of the buoyance force, which is the main reason behind the gas distribution asymmetry in bent tubes [56]. The correlation between the calculated gas distribution asymmetry value and the CFM metering error is presented further in this study.

### 3.3. The CFM Error Correction Based on the Effect of Gas Distribution Asymmetry

As indicated above, the CFM error at multiphase metering has previously been quantified based on the decoupling effect by Equation (1) ([22,23]). However, this quantification is not applicable for many cases, i.e., when the positive CFM error is observed, see [15,23,25,26]. It is suggested that the gas distribution asymmetry should be taken into account for the error estimation as it is able to explain both the positive and negative errors by changing the sign of the gas asymmetry value. Hence, Equation (8) is nominally suggested for the estimation of the CFM error based on the gas distribution effect. However, it should be noted that this equation might be improved further by taking into account the decoupling effect.

(8) $$\varepsilon_{a}=\gamma_{d},$$

where $\varepsilon_{a}$ is a CFM mass flow rate error estimation based on the gas distribution asymmetry effect.

The CFM error estimation based on the gas distribution asymmetry effect ($\varepsilon_{a}$), which is obtained following Equation (8), is presented in Table 4 for the different flow regimes and flowmeters: CMF200, CMF050 and D300. Moreover, the CFM error estimation, which is based on the decoupling effect ($\varepsilon_{d}$), is demonstrated in Table 4. In addition, the CFMs’ errors ($\varepsilon_{e}$), which are observed in the experimental part of this study for CMF200 and CMF050, and in the experimental study by Sultan [23] for D300, are provided in Table 4 for the comparison with the estimated CFM errors. The standard deviations ($\delta_{e}$) of the experimentally observed errors are also included in Table 4. As demonstrated, indeed, the error estimation based on the decoupling effect is simply not able to describe the positive CFM errors and overestimates the negative error by up to two times. This agrees with the observations by Weinstein [15], Liu et al. [25] and Wang et al. [28]. At the same time, the error estimation based on the gas asymmetry effect matches the observed error with a higher precision and can predict both negative and positive CFM errors during multiphase metering at various flow regimes. This is explained by the fact that the gas asymmetry directly influences the CFM tube mass symmetry, which is the foundation of CFM operation and accuracy.

Finally, the experimentally observed CFM errors are corrected based on decoupling and gas asymmetry effects. The corrected errors, which are also presented in Table 3, are calculated following Equations (9) and (10)

(9) $$\varepsilon_{ca}=\varepsilon_{e}-\varepsilon_{a},$$

(10) $$\varepsilon_{cd}=\varepsilon_{e}-\varepsilon_{d},$$

where $\varepsilon_{ca}$ is a CFM mass flow rate corrected error based on the gas distribution asymmetry effect and $\varepsilon_{cd}$ is a CFM mass flow rate corrected error based on the decoupling effect.

As shown in Table 3, the proposed correction, which is based on the nominal gas distribution asymmetry effect, keeps the CFM error in the range of ±10%. This is a significant improvement in comparison with the error without correction, which varies between −17% and 34%. Additionally, the proposed correction is more accurate than the previously available correction, which is based purely on the decoupling effect. The error decrease for this case is up to eight times as the error corrected based on the decoupling effect is between 5% and 68%. At the same time, it should be noted that the proposed correction will benefit from the additional verification on CFMs of different shapes, sizes and manufacturers and different liquids.

## 4. Summary

The gas distribution asymmetry effect on Coriolis flowmeter (CFM) accuracy at multiphase metering is investigated in the present paper. Firstly, the numerical study of gas distribution asymmetry at different flow conditions is conducted. It is demonstrated through a numerical simulation that the gas asymmetry value grows with the increase in the gas volume fraction (GVF) value at the CFM inlet. Additionally, it is shown that the gas distribution asymmetry decreases with the increase in mixture flow rate. This is explained by the influence of buoyancy force, which becomes larger compared to momentum with the decrease in the mixture flow rate and the increase in the GVF at the CFM inlet.

Secondly, the relationship between the CFM multiphase metering error and the gas distribution asymmetry is demonstrated. It is demonstrated that the change in the gas asymmetry is quite close to the change in the metering error with the change in operating conditions. Hence, it is suggested that the CFM error is primarily caused by the gas distribution asymmetry. This is supported by the fact that gas distribution asymmetry causes the overall CFM tube mass symmetry, which is the foundation of the CFM operation. The CFM error correction, which is based on the gas distribution asymmetry effect, decreases the CFM multiphase metering error range to ±10%. This is up to 3.5 times smaller than the non-corrected error range, which is between −17% and 34%. Moreover, this is a significant improvement in comparison with the error corrected based on decoupling, which does not take into account the gas distribution asymmetry effect and is between 5% and 68%.

The investigated asymmetry value depends on the flow rate, gas volume fraction and CFM shape. Hence, the proposed error estimation methodology may be used for any CFM configuration. Additionally, it may be possible to measure the gas volume fractions before and after the CFM tube instead of the simulation to calculate the gas asymmetry value. The appropriate/optimal measurement location is to be determined. Furthermore, the asymmetry value may yet be ascertained as the best measure to quantify flow asymmetry for the CFM operation. There may be a more feasible way to determine the gas distribution asymmetry industrial settings.

Overall, the gas distribution asymmetry in the CFM tube is numerically investigated in the present study. It is shown that this asymmetry depends on the GVF and mixture flow rate values due to the buoyancy force effect. Moreover, the demonstrated relationship between the gas distribution asymmetry and CFM error provides us with an improved understanding of the reasons behind the CFM error during the multiphase flow metering. Finally, the proposed correction of the CFM error, which takes into account the gas asymmetry effect, significantly increases the metering accuracy. The suggested correction might significantly decrease the expenses related to the multiphase flow metering error, which may reach as high as 9% [57]. Therefore, this study improves our understanding of the factors influencing CFM accuracy and serves as the foundation for future studies on multiphase flow metering by CFM.

## Figures and Tables

**Table 1:** Experimentally investigated multiphase flow conditions.

CFM	Case	Water Flow Rate, kg/min	Gas Flow Rate, L/min	$\alpha_{inlet},\;\%$	Flow Regime
CMF200	1	60.8	12.0	16	Stratified
	2		9.0	13	
	3		6.0	9	
	4	100.0	19.5	16	
	5	149.0	15.0	9	Transitional
	6	300.0	15.0	5	Bubbly
	7		26.0	8	
CMF050	11	40.0	7.0	15	Stratified
	12		4.5	10	

**Table 2:** The flow regimes investigated by numerical simulation.

CFM	Case	Water Flow Rate, kg/min	$\alpha_{inlet},\;\%$	Flow Regime
CMF200	1	60.8	16	Stratified
	2		13	
	3		9	
	4	100.0	16	
	5	149.0	9	Transitional
	6	300.0	5	Bubbly
	7		8	
D300	8	182.0	8	Bubbly
	9		12	
	10		16	
CMF050	11	40.0	15	Stratified
	12	40.0	10	

**Table 3:** The investigated flow conditions for the validation of bubbly flow regime [47].

Mixture Flow Rate, kg/s	GVF at the Inlet, %	The Bubbles’ Diameter, mm
29	1.8	2.5 (0.02% GVF)
		5.3 (0.13% GVF)
		6.3 (0.71% GVF)
		8 (0.93% GVF)

**Table 4:** The estimated ($\varepsilon_{a}$ and $\varepsilon_{d}$), experimentally observed ($\varepsilon_{e}$ ) and corrected ($\varepsilon_{ca}$ and $\varepsilon_{cd}$ ) errors of CFMs.

CFM	Case	$\varepsilon_{e},\;\%$	$\delta_{e},\;\%$	$\varepsilon_{a},\;\%$	$\varepsilon_{d},\;\%$	$\varepsilon_{ca},\;\%$	$\varepsilon_{cd},\;\%$
CMF200	1	28	0.1	38	−40	−10	68
	2	34	1.1	33	−30	−1	64
	3	28	0.6	18	−20	10	48
	4	9	0.6	18	−40	−9	49
	5	−5	0.2	3	−20	−8	15
	6	−7	0.2	−1	−11	−6	4
	7	−9	0.3	1	−17	−10	8
D300	8	−12	-	−3	−17	−9	5
	9	−14	-	−4	−27	−10	13
	10	−17	-	−7	−38	−10	21
CMF050	11	12	0.5	3	−35	9	47
	12	5	0.8	2	−22	3	27
